# Arthroscopic Single-Portal Suprapectoral Biceps Tenodesis With All-Suture Anchor

**DOI:** 10.1016/j.eats.2021.10.020

**Published:** 2022-02-08

**Authors:** Abhishek Kannan, Charles J. Cogan, Alan L. Zhang

**Affiliations:** Department of Orthopaedic Surgery, University of California – San Francisco, San Francisco, California, U.S.A.

## Abstract

Tenodesis of the long head of the biceps tendon can be performed through arthroscopic and open techniques with various fixation methods and at different locations on the humerus. Many techniques have been described, with controversy surrounding the advantages and disadvantages of each. In this Technical Note, we describe an all-arthroscopic, intra-articular, single-portal, suprapectoral biceps tenodesis with an all-suture anchor. This technique also allows for suture passage through the biceps tendon before tenotomy to ensure proper maintenance of the length–tension relationship of the biceps musculotendinous unit.

The long head of the biceps (LHB) tendon originates from the supraglenoid tubercle as part of the superior glenoid labrum, and it travels intra-articularly in the glenohumeral joint until exiting the shoulder through the bicipital groove.^1^ The LHB was initially believed to play a dynamic role in humeral head depression and stabilization,^2^^,^^3^ but more recent studies have refuted this role.^4^^,^^5^ Regardless of its biomechanical function, it is a frequent cause of pain in the shoulder.^6^ The reasons for pathology are numerus and include tenosynovitis, tearing, instability, and superior labral tear^7^ (Table 1).

**Table 1:** Indications for LHB Tenodesis

Partial thickness-tear
Instability
Tenosynovitis
SLAP tear
Failed SLAP repair
Clinical examination for LHB pain

LHB, Long head of the biceps.

Nonoperative management of LHB tendon pathology includes rest, ice, nonsteroidal anti-inflammatory drugs, and corticosteroid injection. This is an effective treatment for some patients with isolated biceps pathology, but failure of conservative management or concomitant pathology can necessitate operative intervention. Despite LHB tendon pathology being so common, the current literature on treatment of this pathology remains controversial, as some advocate tenotomy of the tendon, whereas others support tenodesis.^7^, ^8^, ^9^, ^10^ Arguments to support tenodesis include maintaining muscle length, preventing muscle atrophy and deformity, and minimizing cramping.^7^ Both suprapectoral and subpectoral techniques have been described for biceps tenodesis with excellent postoperative outcomes.^6^ Suprapectoral tenodesis can be performed arthroscopically both in or distal to the bicipital groove, whereas the subpectoral technique must be performed through an open incision. Since its early description in the mid-1990s, arthroscopic biceps tenodesis has taken different forms, and the technique has evolved.^11^, ^12^, ^13^ We present an all-arthroscopic technique of intra-articular suprapectoral biceps tenodesis through a single portal with all-suture anchor fixation.

## Surgical Technique (With Video Illustration)

The surgical technique is shown in Video 1. The patient is placed in the beach-chair position with adequate clearance of the posterior shoulder. We prefer to use the beach-chair position for shoulder arthroscopy, but this technique can be performed in the lateral decubitus position as well. The instruments and implants used for this technique are presented in Table 2. The entire upper extremity is prepped and draped in sterile fashion so that the arm may be freely manipulated throughout the procedure. A manual or pneumatic limb-positioning device may be used to hold the arm. A standard posterior portal is established approximately 2 cm inferior and 1 cm medial to the posterolateral corner of the acromion. A 30° arthroscope is inserted into the glenohumeral joint and diagnostic arthroscopy is performed. Intra-articular structures are viewed, and disease of the LHB tendon and superior labral complex is evaluated.

**Table 2:** Equipment and Implants

30° arthroscope
Double-loaded all-suture anchor (No. 2, 1.8-mm VERSALOOP; Depuy-Mitek)
8-mm × 7-mm disposable cannula (Smith & Nephew)
Suture Retrieving Grasper with small penetrating footprint (70° Champion Slingshot; Stryker)
Loop grasper
Arthroscopic knot pusher
Arthroscopic scissors

**Fig 1:**
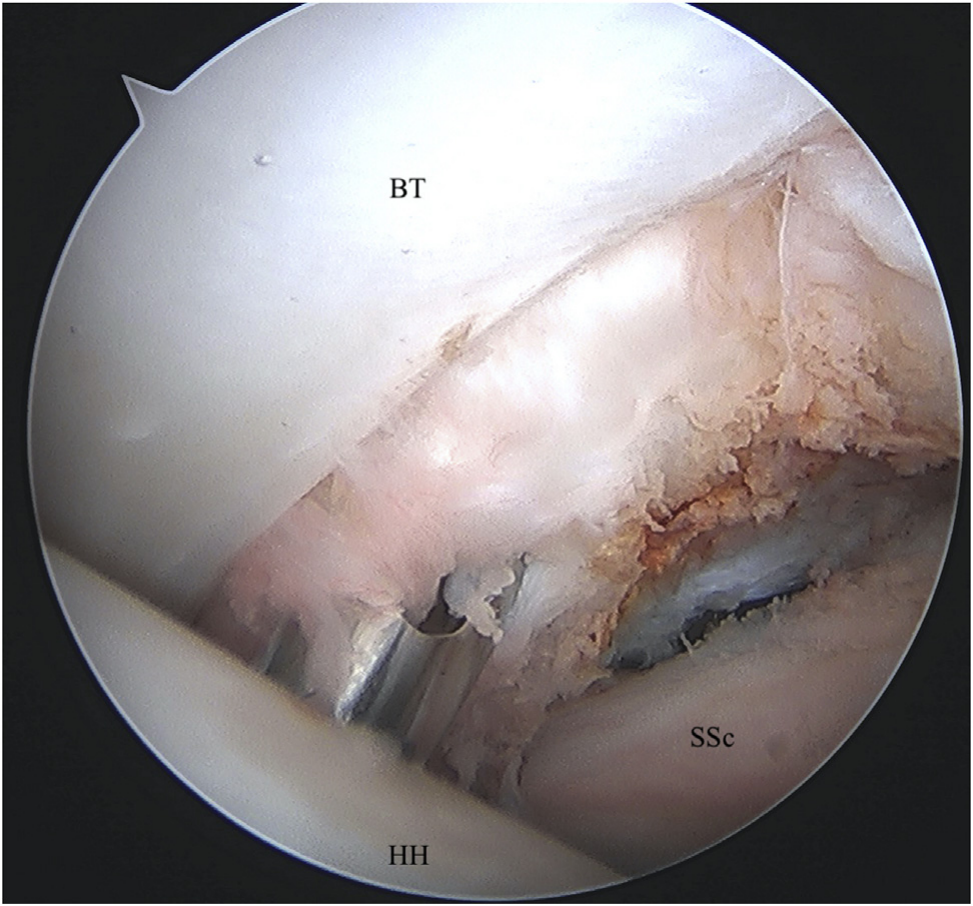
Left shoulder visualized from posterior portal with patient in beach-chair position. A straight drill guide is percutaneously placed in the bicipital groove for anchor placement. (BT, biceps tendon; HH, humeral head; SSc, subscapularis.)

An anterior portal is placed centrally in the rotator interval under visualization and an 8-mm × 7-mm disposable cannula (Smith & Nephew, Andover, MA) is inserted. Establishing the portal in the central or slightly medial aspect of the rotator interval will ensure appropriate trajectory later on for the penetrator through the biceps tendon. Concomitant intra-articular pathology can be addressed through the anterior working portal as needed before biceps tenodesis. The arm is then positioned in approximately 60° of forward flexion with the elbow flexed to 90°. While viewing through the posterior portal, a straight drill guide is introduced percutaneously and placed at the proximal aspect of the bicipital groove (Fig 1). The drill guide should be inserted lateral to the anterior working portal. While holding the drill guide in place, a drill bit is advanced to the appropriate depth with a positive stop. A double-loaded all-suture anchor (No. 2 VERSALOOP 1.8 mm; Depuy-Mitek, Raynham, MA) is then placed into the footprint through the same cannula used for drilling and impacted into bone until the anchor inserter is flush with drill guide and another positive stop is felt.

**Fig 2:**
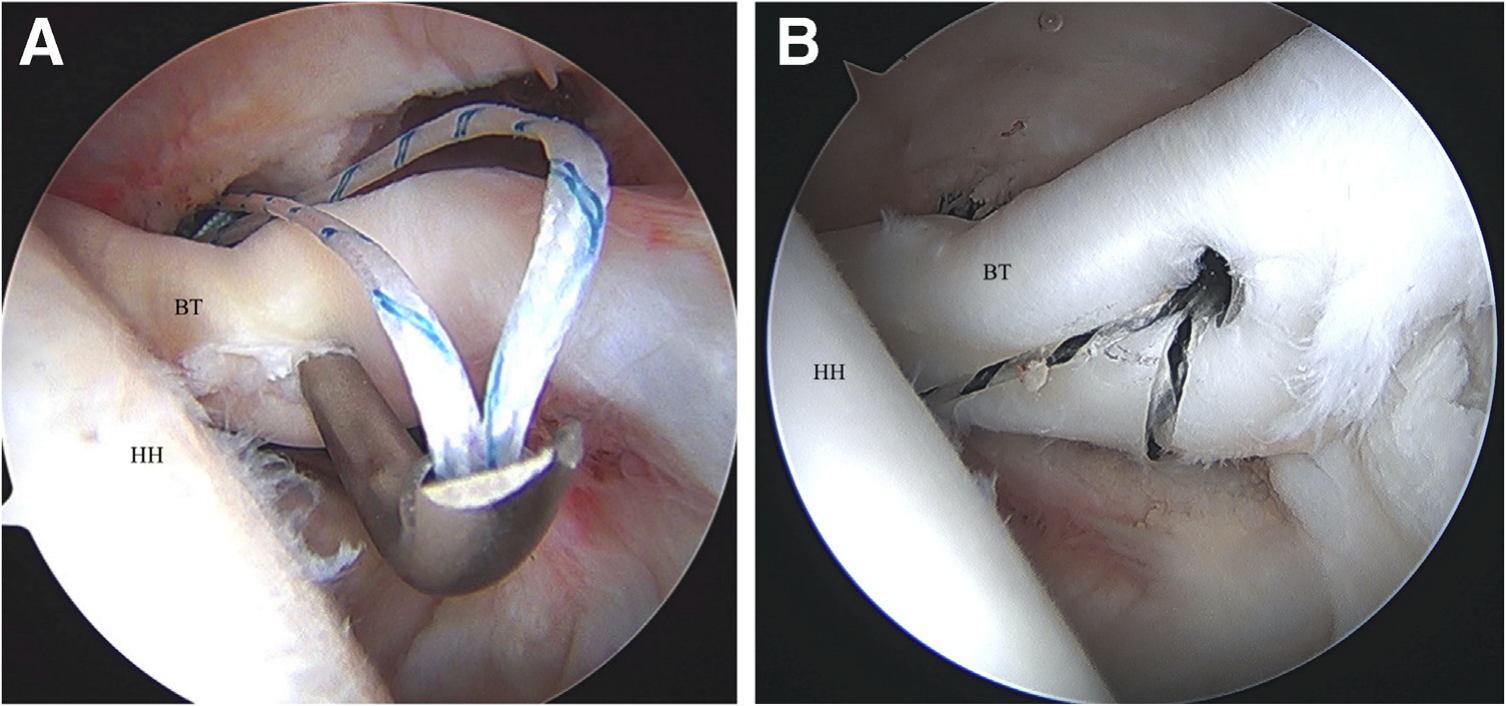
(A) Left shoulder visualized from posterior portal with patient in beach-chair position. A suture passing device (Champion Slingshot) is used to capture and retrieve suture limb through the pierced biceps tendon. (B) Left shoulder visualized from posterior portal with patient in beach-chair position. The suture passing device (Champion Slingshot) is pulled back through the biceps tendon with retrieved suture. (BT, biceps tendon; HH, humeral head.)

**Fig 3:**
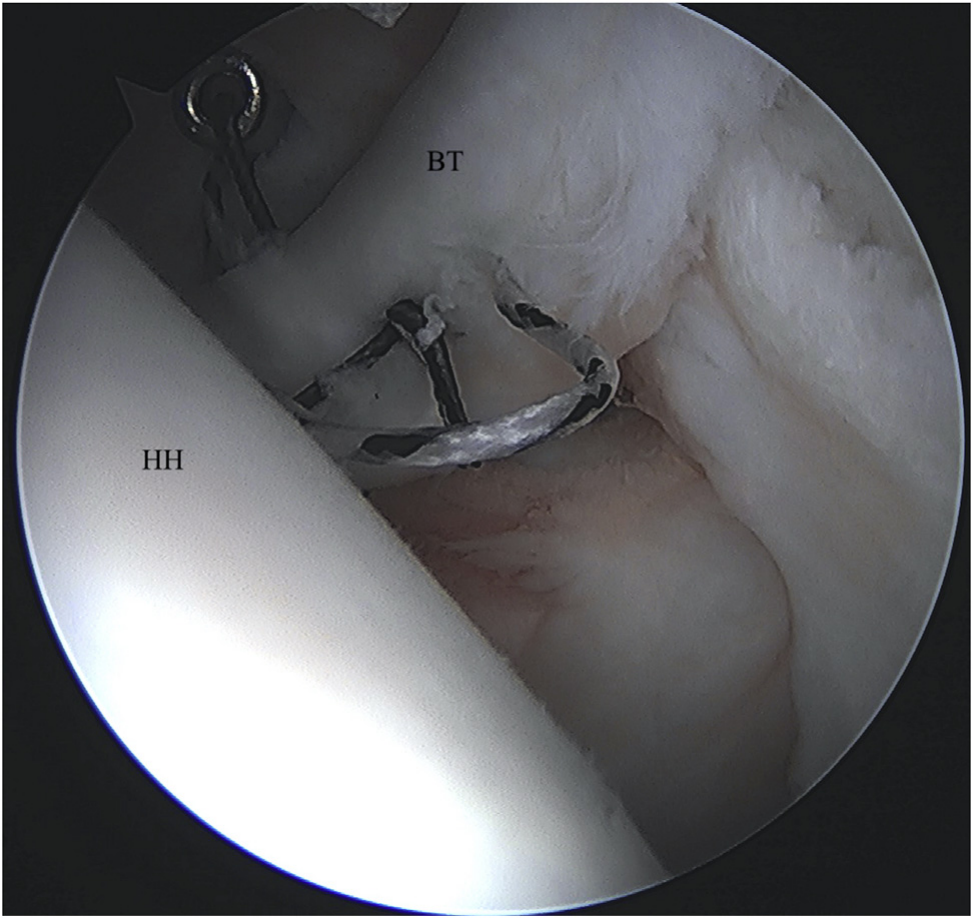
Left shoulder visualized from posterior portal with patient in beach-chair position. Both sets of sutures are passed through the tendon in a simple configuration. (BT, biceps tendon; HH, humeral head.)

Through the anterior working portal, the LHB tendon is pierced using a 70° Champion Slingshot (Stryker, Kalamazoo, MI) suture passing device (Fig 2 A and B). This device is used as the penetrating tip has a small 2-mm footprint that minimizes trauma when it is used to pierce the biceps tendon. The Champion Slingshot is passed through the biceps tendon adjacent to the superior aspect of the bicipital groove where the anchor was placed. This allows for maintenance of the length and tension of the biceps as the sutures are passed while the biceps is still attached to the superior labral anchor complex. A knot-pusher is threaded percutaneously through the path of the suture anchor to deliver one of the suture limbs to the Champion Slingshot for retrieval. The Champion Slingshot is closed and pulled back out the working cannula to complete passage of the suture through the biceps tendon. This step is repeated with the other suture from the double-loaded anchor to create 2 simple suture passes through the biceps tendon (Fig 3).

**Fig 4:**
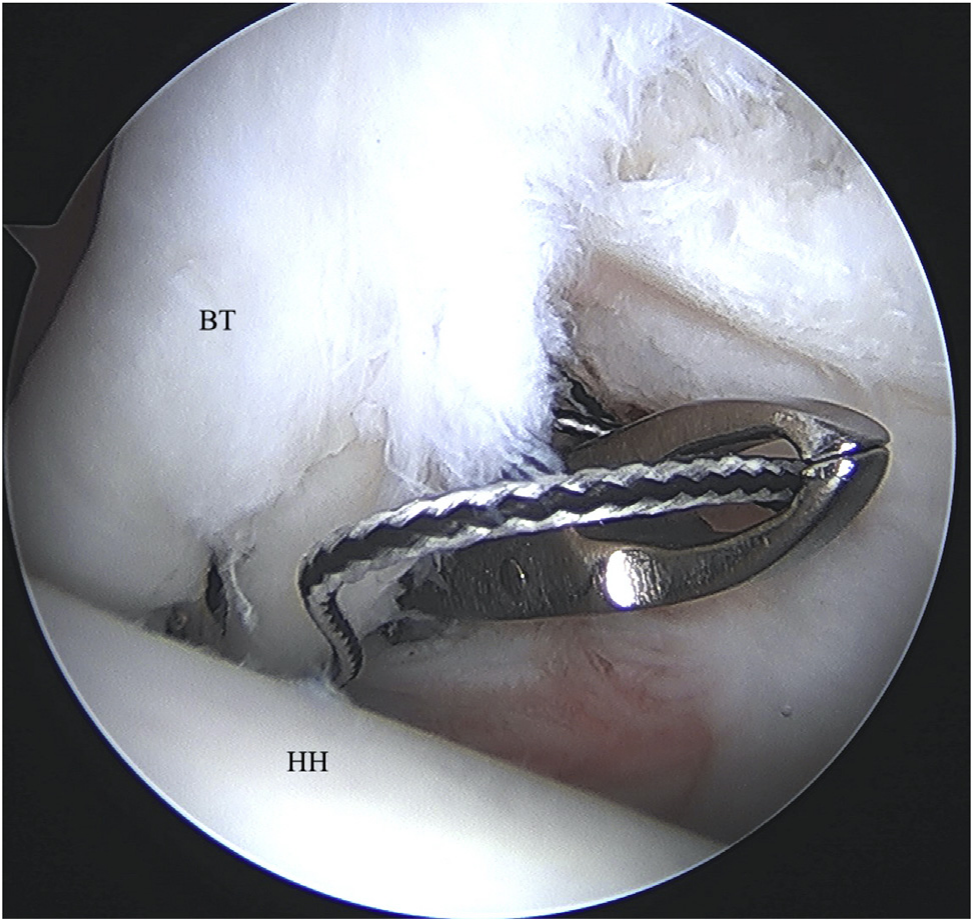
Left shoulder visualized from posterior portal with patient in beach-chair position. The corresponding suture limbs are retrieved from the anterior working portal, and arthroscopic knots are tied. (BT, biceps tendon; HH, humeral head.)

After retrieval of the corresponding limb of each passed suture through the anterior working cannula (Fig 4), arthroscopic knots are then tied down using alternating half-hitches to secure the tendon to the humerus prior to tenotomy. These steps are completed in sequence and instrumented through a single anterior portal while viewing intra-articularly from a standard posterior portal. As the tenodesis is completed before complete biceps tenotomy from its superior labral anchor, the length-tension relation of the biceps musculotendinous unit is maintained.

**Fig 5:**
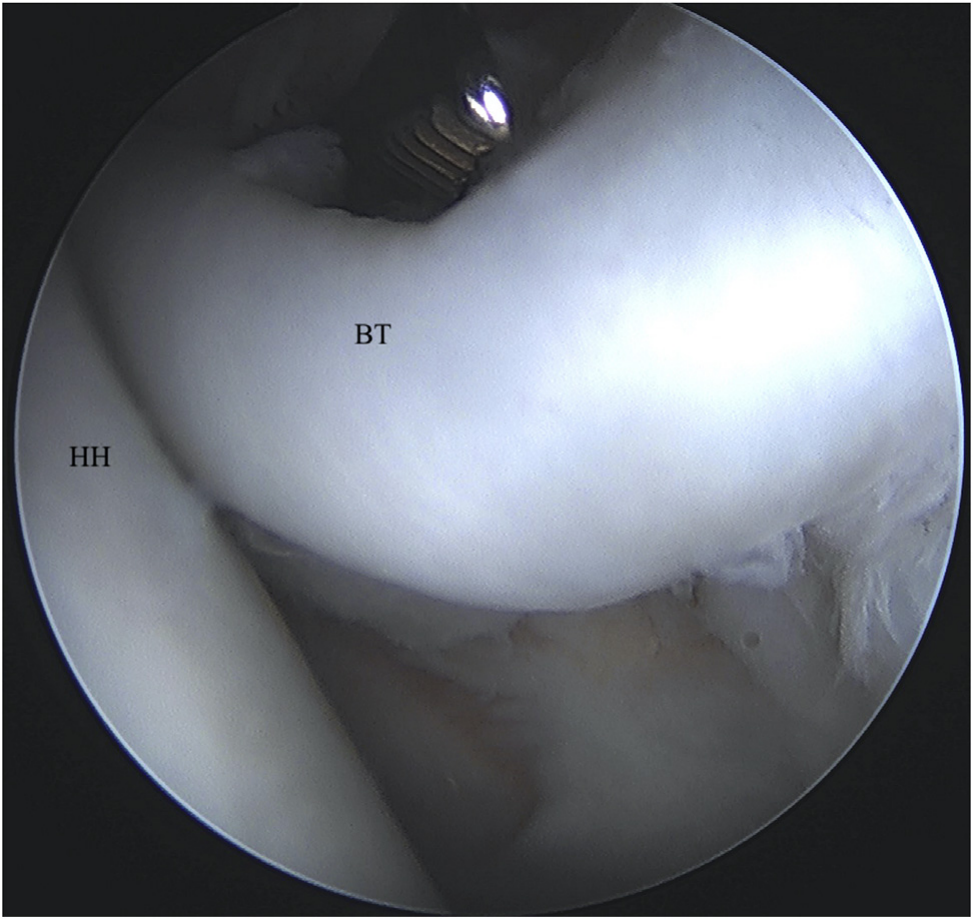
Left shoulder visualized from posterior portal with patient in beach-chair position. The biceps tenotomy is completed with arthroscopic scissor or basket punch. (BT, biceps tendon; HH, humeral head.)

**Fig 6:**
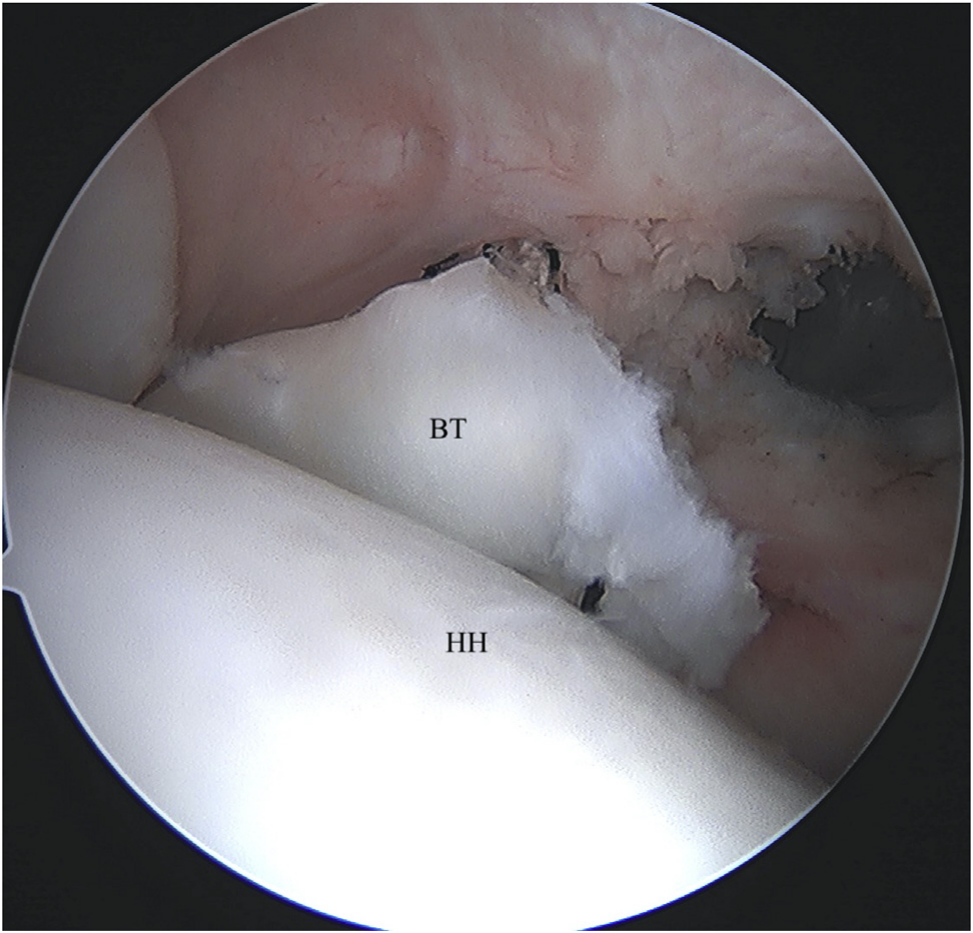
Left shoulder visualized from posterior portal with patient in beach-chair position. A completed intra-articular, suprapectoral biceps tenodesis is viewed from the standard posterior portal. (BT, biceps tendon; HH, humeral head.)

Once the biceps tenodesis is confirmed to be structurally sound with a probe, the residual intra-articular portion of tendon is released from a site proximal to the anchor using arthroscopic scissors (Fig 5) and the remaining stump is excised at the anchor point adjacent to the superior labrum using a basket punch and shaver. The arm and elbow are then taken through a full range of motion to ensure stability of the tenodesis (Fig 6). Pearls and pitfalls for this technique are listed in Table 3.

**Table 3:** Pearls and Pitfalls

Pearls	Pitfalls
Place working portal slightly medial in rotator interval for optimal trajectory to pierce tendon with suture passing device (Champion Slingshot)	Far lateral placement of working portal in the rotator interval Inadequate distance between working portal and percutaneous anchor
Percutaneous incision lateral to the anterior portal to reach apex of bicipital groove for appropriate tenodesis site	Soft-tissue bridge formation during threading of knot pusher
Adequate spread between working portal and percutaneous anchor for knot pusher to reliably deliver suture limb to suture passing device	Repeated passes through tendon with suture passing device can damage integrity of tendon

## Discussion

We present an all-arthroscopic biceps tenodesis technique that places the tenodesis site proximal to the bicipital groove in the suprapectoral region that can be performed from the glenohumeral joint. The majority of studies comparing arthroscopic with open tenodesis have demonstrated no significant differences regarding postoperative pain, patient satisfaction, or outcomes scores.^14^, ^15^, ^16^, ^17^, ^18^

Complications associated with all forms of biceps tenodesis include length–tension mismatch, loss of fixation, persistent pain, shoulder stiffness, infection, hematoma, neurologic injuries, vascular injuries, and reflex sympathetic dystrophy.^19^ Regarding postoperative complications, Werner et al.^20^ found the arthroscopic approach resulted in a greater incidence of postoperative stiffness. While open tenodesis techniques have potential complications associated with open surgery,^21^ including blood loss, wound infection, nerve injury, and cosmetic deformity from scar, overall complication rates remain low.^22^, ^23^, ^24^

The literature remains inconclusive regarding optimal site of tenodesis, that is, above (suprapectoral) or below (subpectoral) the insertion of the pectoralis major tendon. Described suprapectoral arthroscopic techniques have included tenodesis sites proximal, within, or distal to the bicipital groove.^11^^,^^14^^,^^25^^,^^26^ In a systematic review and meta-analysis including 7 retrospective studies with 409 patients, 187 treated with suprapectoral tenodesis, and 222 with subpectoral tenodesis, van Deurzen et al.^6^ evaluated outcome measures including American Shoulder and Elbow Surgeons (ASES), Constant–Murley Score, visual analog scale, bicipital groove pain, and Popeye deformity. The mean difference in ASES score, while statistically significant in favor of subpectoral tenodesis, did not meet clinical relevance. They found no significant differences among the remaining outcome measures between the 2 methods.

Fixation options described include, but are not limited to, sutures, suture anchors, interference screws, and cortical buttons.^16^^,^^27^, ^28^, ^29^, ^30^, ^31^ When evaluating clinical outcomes of the 2 fixation methods in the setting of biceps tenodesis, Millett et al.^32^ found no difference in ASES, visual analog scale, and modified constant scores between all-suture anchors and interference screws over 1-year postoperatively. Frank et al.^33^ evaluated torsional strength and fracture risk using all-suture anchors, conventional suture anchors, and interference screws. Interference screw constructs fractured through the 8-mm pilot hole created with significantly lower rotational displacement compared to the anchor-based constructs. In total, 57% of fractures traversed the 2.9-mm hole created for conventional anchors, compared to 29% in the 1.9-mm pilot hole for all-suture anchors. These findings suggest smaller holes likely reduce the risk of postoperative fracture under torsional stress. The benefits of an all-suture suture anchor construct as described in our technique include biomechanical benefits offered by traditional screw or anchor fixation but in a low-profile construct. The small diameter tunnel for the all-suture anchor leads to inherent bone preservation, less trauma to the humeral cortex, and diminished fracture risk.

Arthroscopic suprapectoral biceps tenodesis has been described with use of various fixation methods and alternative exposures and preparation.^11^^,^^25^^,^^34^^,^^35^ Our method of an all intra-articular suprapectoral tenodesis with all-suture anchor depicts ease of use, reliability of exposure, and straightforward preparation, while minimizing risk of adverse events or complications. The all intra-articular technique, as we have described, reduces fluid extravasation and eliminates the need for preliminary subacromial decompression^34^ or subdeltoid bursectomy.^25^ The biceps is tenodesed under direct visualization from a standard posterior arthroscopic viewing portal, an anchor is placed adjacent to the articular margin at the proximal exit of the biceps groove, and all steps are completed without manual tensioning of the tendon. Tenotomy performed after completion of tenodesis ensures biceps length-tension relationship is maintained. A 1.8-mm all-suture anchor is low-profile, preserves proximal humerus bone stock, and diminishes risk of fracture. Use of a penetrating device with both suture deployment and retrieval mechanisms allows performance of tendon capture and placement to be completed through a single portal. Additional steps of tendon tagging, passage, and retrieval are eliminated. A summary of the strengths and limitations with this technique is presented in Table 4.

**Table 4:** Strengths and Limitations

Strengths
Minimize incisions and capsular disruption
Maintains length-tension relationship of LHB
Requires simple equipment
Limitations
Does not address groove pathology
Risk of tendon damage from repeated penetration of suture passer

LHB, Long head of the biceps.

## Conclusions

We present a technique for arthroscopic, suprapectoral, intra-articular biceps tenodesis through a single anterior portal with an all-suture anchor. This technique allows for suture passage through the biceps tendon before tenotomy to ensure proper maintenance of the length-tension relationship of the biceps musculotendinous unit and offers the benefits of arthroscopic procedures, including an overall less invasive procedure, better cosmesis, and decreased blood loss.

## Supplementary Data

ICMJE author disclosure forms

Video 1 Video presentation of the surgical technique for arthroscopic single-portal suprapectoral biceps tenodesis with an all-suture anchor.
